# A mathematical kinetic model of memory in *Bacillus subtilis* spore germination

**DOI:** 10.1016/j.isci.2026.115864

**Published:** 2026-04-22

**Authors:** Chris G. de Koster, Xiuping Lin, Yong-qing Li, Stanley Brul, Leo J. de Koning, Peter Setlow

**Affiliations:** 1 Mass Spectrometry of Biomacromolecules, Swammerdam Institute for Life Sciences, University of Amsterdam, Science Park 904, Amsterdam 1098 XH, the Netherlands; 2 Molecular Biology and Microbial Food Safety, Swammerdam Institute for Life Sciences, University of Amsterdam, Science Park 904, Amsterdam 1098 XH, the Netherlands; 3 Department of Physics, East Carolina University, Greenville, NC, USA; 4 Department of Molecular Biology and Biophysics, UConn Health, Farmington, CT, USA

**Keywords:** Biological sciences, Microbiology, Microbial physiology, Microorganism

## Abstract

Dormant *Bacillus subtilis* spores germinate through interaction of germinants with germinant receptors (GRs). Subsequently, GR signals are transduced to SpoVA protein channels, and opening these channels leads to calcium dipicolinic acid (CaDPA) release and completion of germination. Spores exhibit memory in germination, as spores given a short germinant pulse more readily respond to a second pulse. We developed a mathematical model to identify the minimal network crucial for germination kinetics leading to memory of germinant exposure, and reproducing experimental double germinant pulse germination curves. Analysis of the reconstructed network indicates that a minimal set of inactive and active GRs and a SpoVA channel in three states—closed inactive, closed active, and open—is needed to reproduce memory. Spore germination memory is introduced in the network by GR’s activation and deactivation rates and the interplay between activation of closed SpoVA channels and their rates of opening and closing.

## Highlights

• Spores germinate via germinant receptors sensing environmental cues
• Spores show memory: stronger response to a second germinant pulse
• Model reproduces germination memory using GR states and SpoVA channels
• Simulations reveal key roles of CaDPA channel states in memory dynamics

## Graphical abstract

**Figure:**
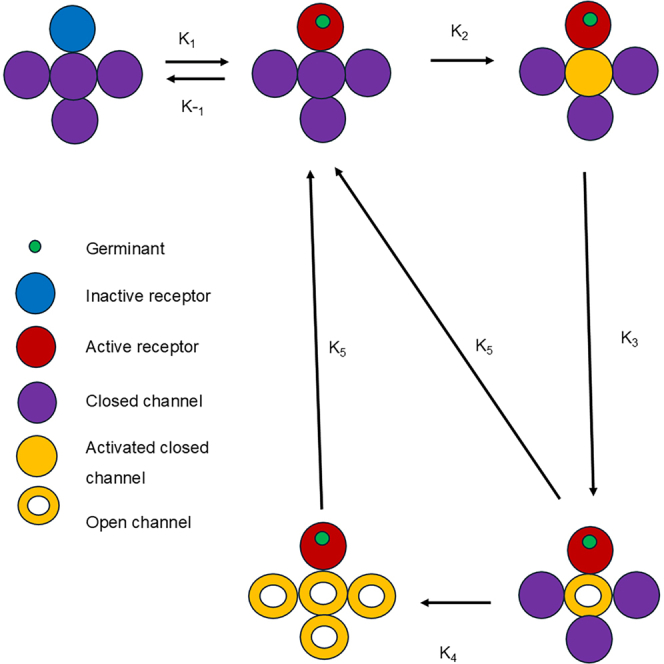

## Introduction

Spores of bacteria *Bacillus subtilis* are formed in sporulation and are dormant and with an overall structure quite different from that of a growing cell.^1^ From the outside in, spores contain an exosporium in some species (but not *B. subtilis* spores), then a layer of protein termed the coat, then an outer membrane above the spore’s peptidoglycan cortex that contains muramic acid lactam, next a layer of germ cell peptidoglycan with no muramic acid lactam, then an inner membrane (IM) encircling the spore core that contains all nucleic acids; most spore enzymes and a high level of the 1:1 chelate of Ca^2+^ with pyridine-2,6-dicarboxlic acid (CaDPA, ∼10% of spores core dry wt), as well as low levels of core water, as low as 20% of core wet weight.^1^ Spores are extremely resistant to all manner of treatments, have no ATP but can survive harsh environmental conditions for many years.^1^^,^^2^^,^^3^ However, when nutrients become available and conditions are favorable for growth, spores can germinate and outgrow into vegetative cells via a sequence of molecular processes and signaling through pathways and networks.^4^^,^^5^^,^^6^^,^^7^ In the 1^st^ stage of germination the spore probes its environment for the availability of nutrient germinants. Upon interaction of germinants with germinant receptors (GRs) in the IM, spores germinate. *Bacillus* GRs generally have 3 subunits, A, B, and C, and these are gated ion channels with other ion channels participating.^8^^,^^9^^,^^10^^,^^11^^,^^12^ The binding of germinants, in particular to GR’s B subunits, leads to release of the spore’s huge depot of (CaDPA). This release then triggers the activation of two cortex-lytic enzymes (CLEs), CwlJ and SleB. In the 2^nd^ stage of germination, the CLEs hydrolyze the large spore cortex peptidoglycan (PG) layer, as these enzymes recognize muramic acid lactam which is only present in cortex PG.^1^^,^^2^^,^^3^^,^^4^ Cortex degradation then allows core expansion with full hydration of the spore core which allows resumption of normal metabolism, macromolecular synthesis, and finally outgrowth into a vegetative growing cell.

The GR protein assemblies GerA, GerB, and GerK in *B. subtilis* are each encoded by homologous tricistronic operons *gerA*, *gerB* and *gerK*.^1^^,^^2^^,^^3^^,^^4^ GR subunits A and B are integral membrane proteins that are embedded in the spores’ IM through multiple predicted α helices. Subunit C is a peripheral membrane protein, attached to the IM by a diacylglycerol anchor.^13^ The GerA GR responds to L-alanine or L-valine, and the GerB and GerK GRs cooperatively respond to a mixture of L-asparagine, D-glucose, D-fructose, and K^+^ (AGFK). A GerB variant, GerB∗, germinates in response to L-asparagine alone. Average wild-type GR spore copy numbers have been determined by western blotting and are ∼2,500 total GRs/spore, with GerA at ∼1,100 molecules/spore; and GerB and GerK each at ∼700 molecules/spore.^14^ As expected, GR levels in spores affect the germination rate, and GR overexpression increases rates of spore germination by these GRs.^15^ All *B subtilis* GRs co-localize in the spores’ IM in a cluster termed the germinoso.^16^ and germinosomes have also been found in other *Bacillus* species.^17^ The GerD protein co-localizes with GRs in the germinosome and is essential for germinosome formation, presumably acting as a scaffold, although the germinosome structure has not been determined. GerD is also essential for normal GR-dependent spore germination, as *gerD* spores germinate extremely poorly with L-valine via GerA or AFGK via GerB plus GerK.^18^ Like GR C subunits, GerD is also likely attached to the IM through an N-terminal diacylglycerol anchor attached to an N-terminal cysteine residue.^18^

The signal generated upon GR activation, likely involving cations released from the spore core, is transduced to SpoVA proteins that make up a channel for CaDPA in the spore IM. However, the signaling mechanism is not fully understood, and no proteins that act in between the GRs and the SpoVA channel protein complex have been identified. The *B. subtilis spoVA* operon encodes SpoVAA, SpoVAB, SpoVAC, SpoVAD, SpoVAEb, SpoVAEa, and SpoVAF proteins. SpoVAA, SpoVAB, SpoVAC, SpoVAEb, and SpoVAF are predicted to be integral membrane proteins based on their primary sequences, and SpoVAD and SpoVAEa are hydrophilic, soluble proteins that are on the outer IM surface.^19^^,^^20^ The copy numbers of SpoVAD and SpoVAEa have been determined by western blotting as ∼6,000–6,500 and 750 molecules/spore, respectively.^19^^,^^20^ SpoVAC alone functions as a mechanosensitive channel for small molecules such as CaDPA, with several treatments including membrane tension and the non-GR-dependent germinant dodecylamine opening this channel *in vivo* and *in vitro*.^4^^,^^21^^,^^22^ While the arrangement of SpoVA proteins in a supramolecular structure is not known, it seems most likely that the SpoVA proteins are subunits of a protein assembly in the spore’s IM that constitutes a channel for CaDPA movement into the developing spore during sporulation and out of the germinating spore.^23^

In contrast to GRs which are in the multiprotein germinosome, SpoVA proteins are more evenly distributed over the spore IM.^16^ Opening of the CaDPA channel is triggered not only by GR activation but also by dodecylamine or hydrostatic pressures of ≥4,000 atm.^4^ Notably, while there is slow leakage of CaDPA from spores after germination is begun,^24^ almost all CaDPA is released in a very short time and in parallel with water uptake into the core.^4^

In the 2^nd^ germination stage, CaDPA activates the CLEs which hydrolyze the cortex PG. CwlJ is activated first by CaDPA released from the spore core and SleB is activated only after substantial CaDPA release, perhaps by changes in cortex PG structure accompanying CaDPA release and water uptake. CwlJ can also be activated by exogenous CaDPA. The structure of the dormant spore cortex PG and physical effects due to elastic and contractile properties of the cortical PG sacculus presumably affect the susceptibility of opening of SpoVA channels, as does crosslinking in the spore coat which continues even after spores are released from sporangia.^25^^,^^26^ Cortex hydrolysis degrades the cortex PG macromolecular network restricting expansion of the spore core, thus enabling further uptake of water. As core water content increases, eventually full core hydration allows onset of metabolic reactions including biosynthesis of molecules needed for cell growth.

Spore germination is a heterogeneous process, as in populations, some spores germinate rapidly and some extremely slowly.^27^^,^^28^^,^^29^^,^^30^^,^^31^ This heterogeneity is primarily due to a variable time period between the application of a germinant stimulus and the onset of rapid CaDPA release from the spore core. This time interval is denoted as the lag time *T*_lag_; all CaDPA is released by *T*_release_, and cortex PG hydrolysis and core swelling are complete by *T*_lysis_. Spores that are exposed to germinant molecules for a short time interval can be committed to germination at time *T*_c_ even when the germinants are removed and further binding to GRs is blocked^28^^,^^29^^,^^30^^,^^31^; the slow leakage of 15%–25% of CaDPA begins at or close to *T*_c_, and this slow release ends at *T*_lag_, when rapid CaDPA release begins.^24^ *T*_lag_ values are decreased by spore heat activation, higher GR levels and higher levels of GR-dependent germinants up to saturation.^19^ Spores with ∼4-fold higher SpoVA copy numbers have lower *T*_lag_ values in GR germination than those for wild-type spores.^15^ The times needed for rapid release of all CaDPA remaining at *T*_lag_ (Δ*T*_release_ = *T*_release_-*T*_lag_) (0.5–3 min), and for cortex hydrolysis and core swelling (Δ*T*_lysis_ = *T*_lysis_ - *T*_release_) (10–20 min) are generally shorter than *T*_lag_. GR levels and nutrient germinant concentrations do not appreciably affect Δ*T*_release_ or ΔT_lysis_ values. However, loss of the CwlJ CLE, but not SleB, markedly decreases rates of CaDPA release during spore germination.

Notably, spores were found to exhibit memory of prior germinant exposures.^30^^,^^32^^,^^33^ Thus spores given a brief exposure to a germinant that activates the GerA or GerB∗ GRs responded more readily to a 2^nd^ short germinant pulse. However, the response to the 1^st^ germinant pulse diminished when the time interval in between pulses was increased,^30^^,^^32^ indicating that there is memory in the germination system. Furthermore, nonnutrient germinants such as GR-independent CaDPA or dodecylamine, which do not require GRs, exhibit memory either alone or in combination with nutrient germinants,^32^ suggesting that the SpoVA channel proteins are the most likely candidates for memory storage while GRs are the candidates for memory generation. It has been proposed that spore memory is primarily stored in metastable states of SpoVA proteins,^32^ and/or that the spore’s electrochemical potential plays a role in enabling this memory.^34^ However, the involvement of electrochemical potential in the memory of germinant exposure appears unlikely as it was found that Thioflavin-T cannot report on electrochemical potential of germinating spores,^33^ but mechanisms underlying spore germination memory are not clear.

The pioneering work on mathematically modeling germination kinetics and heterogeneity was conducted by Woese et al. in 1968.^35^ Spore germination was assumed to result from the accumulation of an unknown substance P, with its production rate being proportional to the number n of activated germination “enzymes”. By assuming that a threshold amount of P was required for initiating germination, it was predicted that time-to-germination of a spore with n active enzymes is proportional to 1/n. Furthermore, by assuming that the fraction of spores with n active enzymes in a population follows a Poisson distribution, this simple model explained spore heterogeneity and germination kinetics. Recently, Woese’s work was extended to a comprehensive mathematical model to describe heterogeneity, commitment, memory, and kinetic CaDPA release of spore germination,^36^ in which each spore in a population possesses m functional GRs following a statistical distribution, the binding of a nutrient germinant activates a number of GRs and produces a time-dependent germination trigger signal. It was assumed that when the trigger signal is above a critical value, the spore is committed to germinate. This commitment results in the opening of the SpoVA channel, leading to the release of CaDPA and full spore germination.^36^ This model’s predictions fitted very well with the experimental data of heterogeneity, commitment, memory, and CaDPA release at the single spore level. However, the biochemical details for the trigger signal or germination substance P remain unclear.

In the current work, we have attempted to develop a mathematical model to identify a minimal reconstructed molecular network that is crucial for germination kinetics and memory. We consider a minimal set of GRs in two states—inactive and active, and a SpoVA channel in three states—closed inactive, closed active, and open. When the number of SpoVA proteins in the open active state reaches a threshold level (e.g., 50 copies/spore used in this paper), the channel is largely opened for rapid CaDPA release. Numerical simulation reveals that memory can reside with GRs and/or be stored in the CaDPA channel complex, which is consistent with the previous proposal that spore memory is stored in metastable states of SpoVA proteins.^32^ Obviously, this model must be able to predict both the heterogeneity of *T*_lag_ and the percentages of spore germination in 1^st^ and 2^nd^ germinant pulses. In addition, with computer simulations, we can identify network topologies that exhibit minimal deviation between predicted and experimental data and reveal reaction dynamics of molecular processes between GR activation and rapid CaDPA release. Finally, analysis of the optimized model could shed light on the question of how the network’s molecules and their interactions determine its biological function.

## Results

### Spore germination model

In our modeling strategy, we follow a top-down approach. First, we develop an overall mathematical model and then we dissect the mathematics into a set of mass action kinetics based differential equations based on the components of the germinosome and the SpoVA channel which must somehow interact. As a boundary condition, we opted for the most parsimonious number of building blocks, and that the model must be able to recreate published experimental spore memory data.^32^ One common premise for commitment in molecular networks is a bistable switch.^37^ In such bistable systems two stable states coexist, and thus spores are dormant or are committed to germinate.^38^ Upon activation, the system is irreversibly committed to one state. A condition for bistability is non-linear dynamics involving positive feedback in which a molecular species upregulates its production or autoactivates its function, e.g., channel opening, a mechanism to filter out noise and a mechanism to prevent numeric explosion of the upregulated or activated species.^38^^,^^39^ A simple deterministic molecular kinetic model for the autoactivation and deactivation rates of the state variable *X* commonly used to model bistability is given in Equation 1.^40^^,^^41^

(Equation 1) $$\frac{dX}{dt}=P+k_{s}\frac{X^{n}}{X^{n}+\theta^{n}}-k_{d}X\text{.}$$

In this equation, *X* denotes the concentration (molecules/spore) of a crucial state of a germination protein, *k*_s_ is its maximum production rate, *n* represents cooperativity of variable *X*, *θ* ^n^ sets the value at which the rate of production of *X* is half its maximum value, *k*_d_ is the decay rate of protein *X*, and *P* is a basal production rate.^40^^,^^41^^,^^42^ Positive feedback is introduced by cooperative action of the system variable *X* for *n* > 1. The multistabilty of Equation 1 depends on the settings of the various parameters. There are some settings in which *X* gradually increases upon an inductive stimulus and there is a parameter subspace where *X* exhibits a bistable response. In the latter case, there are two stable states, one at low *X* and one at high *X* and an unstable state in between. Fluctuations of *X* mediated by *P* around the lower stable state (state 1) but not progressing to the metastable state fall back to the lower stable state. Fluctuations of *X* through *P* surpassing the unstable state are auto-activated and increase to the stable state 2 at high *X*. Fluctuations in *X* above the high stable state fall back to stable state 2, thereby preventing a runaway of variable *X* to infinity. Equation 1 is thus an autocatalytic off-on switch with parameter subspace for bistability and is the core of our model.

Starting from this equation, we designed an input section, signal transduction, and finally describe SpoVA channel opening kinetics to estimate *T*_lag_ times in germination. The molecular wiring of the reconstructed molecular network of the model is given in the graphical abstract. The molecular network is composed of GR proteins that are activated by a germinant, and channel proteins are in a closed, activated closed, and an open state and undergo mutual interactions. In our model, we do not differentiate between GerA, GerB, and GerK GRs and thus do not differentiate between germinants. GRs can be in an inactive *R*_i_ state or an active *R*_a_ state, and germinant *S* binding converts an *R*_*i*_ GR to *R*_*a*_ with a rate constant *k*_*1*_. Low copy protein numbers in individual cells in bacterial populations usually vary according to a gamma distribution.^43^ Measurements of levels of GFP-labeled GRs in spores have shown that GR copy numbers also vary according to a gamma distribution function.^43^^,^^44^ Thus, the distribution of total levels of *R*_i_ in spores in the absence of germinant is given by the probability density (gamma) function

(Equation 2) $$f\left(R_{i};a,b\right)=R_{i}^{a-1}\frac{e^{-R_{i}/b}}{b^{a}\Gamma \left(a\right)}\text{,}$$

in which Γ(*a*)=(*a*-1)!. *a* is the shape parameter, and *b* is an inverse scale parameter. The distribution mean *E*(*R*_i_) = *ab* and the variance *Var*(*R*_i_) of the distributions is *ab*.^2^ The skewness of the distribution is equal to 2/*a*^½^. The skewness depends only on the shape parameter *a* and approaches a normal distribution when approximately *a* > 10.

Germinant binding in our model is reversible and dissociation takes place with rate constant *k*_-1_. Equation 3 and Equation 4 describe rates of formation of active GR molecules *R*_*a*_ and rates of depletion of the inactive GR population *R*_*i*_. In contrast to GRs which we propose exist in only two states, *R*_i_ and *R*_a_, we need a SpoVA channel that can adopt three states. While there is no experimental proof for the latter assumption, a model with two SpoVA states does not reproduce experimental germination curves (data not shown). The SpoVA channel *C*_c_ is closed and inactive, the channel *C*_*ca*_ is closed and in an activated state, or the channel *C*_*o*_ is open and in an activated state. Interaction of activated GRs *R*_*a*_ with closed SpoVA channels *C*_*c*_ activates the latter (Equation 5). We assume a direct interaction, although there are no conclusive experimental data about signaling from activated GRs to the SpoVA channel. However, yeast two hybrid experiments suggest that there is interaction between *B. subtilis* GerA proteins and several SpoVA proteins.^45^ The function of GerD is ignored in the model. A premise for commitment in molecular networks is a bistable switch with delay. Bistability is introduced here through intersection of a production function comprising a Hill function with *n* > 1 and a linear decay function in Equation 7. The production function describes the rate of opening of SpoVA channels. Cooperativity of open SpoVA channels also contributes to the opening rate. This means that open SpoVA channels (C_*o*_) have multiple mutual interactions which enhance the opening rate of closed channels. A possible mechanism for this cooperativity is that the subthreshold open SpoVA channels cause slow CaDPA release prior to T_lag_,^24^ the passage of this intracellular CaDPA activates the CLE CwlJ and this enzyme’s action stimulates further opening of the SpoVA channel for CaDPA efflux, leading to an increase in C_o_ until the threshold is reached. Slow leakage of CaDPA prior to T_lag_ from individual *Bacillus* spores has been experimentally observed by Wang et al.^24^ Cooperativity of SpoVA channel gating is introduced through a Hill function with apparent dissociation constant *θ* and coefficient *n*. The decay function describes closure of channels with reaction rate –*k*_5_*C*_*o*_. Channel closure is first order in the concentration of open channels. Upon closure the closed channel falls back to the inactive ground state *C*_c_.

(Equation 3) $$\frac{dR_{i}}{dt}=-k_{1}SR_{i}+k_{-1}R_{a}$$

(Equation 4) $$\frac{dR_{a}}{dt}=k_{1}SR_{i}-k_{-1}R_{a}$$

(Equation 5) $$\frac{dC_{c}}{dt}=-k_{2}R_{a}C_{c}-\frac{{k_{4}C}_{o}^{n}}{C_{o}^{n}+\theta^{n}}+k_{5}C_{o}$$

(Equation 6) $$\frac{dC_{ca}}{dt}=k_{2}R_{a}C_{c}-k_{3}C_{ca}$$

(Equation 7) $$\frac{dC_{o}}{dt}=k_{3}C_{ca}+\frac{k_{4}C_{o}^{n}}{C_{o}^{n}+\theta^{n}}-k_{5}C_{o}$$

In these equations, *S* is germinant concentration, *R*_*i*_ inactive GR, *R*_*a*_ active GR, *C*_*c*_ closed SpoVA channel, *C*_*ca*_ activated closed SpoVA channel, and *C*_*o*_ open SpoVA channel. The units of the GR and SpoVA variables are numbers of the various complexes/spore. Constants *k*_1_, *k*_-1_, *k*_2_, *k*_3_, *k*_4_, and *k*_5_ are rate constants, *n* is a Hill coefficient, and *θ* ^n^ sets the value at which the production rate in the second term is half its maximum value. The final stage of the model is SpoVA channel opening. Whether core CaDPA is exported by concentration differences and/or an electrochemical gradient of ions inside and outside the inner membrane, CaDPA efflux will be proportional to the number of open SpoVA channels, and we assume that *T*_lag_ is determined by a minimal number of open SpoVA channels needed for rapid CaDPA release. It is known that CaDPA activates the CLE CwlJ and this enzyme’s action stimulates CaDPA efflux. However, CwlJ activation and its effect on the SpoVA channel opening rate is not yet a part of the model, and we expect that this effect will become significant only after *T*_lag_. If there is a contribution of CwlJ activation to SpoVA opening than this will lead to a minor shift of *T*_lag_. In our computer model, we use a cycle of 500 numeric simulations to solve the set of coupled ordinary differential equations. In each simulation, we predict the number of active open SpoVA channels as a function of time and we determine for each simulation the time of opening, *T*_open_, of SpoVA channels. This time point corresponds with the time point at the beginning of fast CaDPA release^28^ and is related to *T*_lag_ through *T*_lag_ = *T*_*open*_ – *T*_block_, where *T*_block_ is the time of the end of a germinant pulse. We define *T*_open_ as the time point in a simulation where *C*_o_ exceeds 50 copies/spore. This number is arbitrarily selected as experimental data are lacking.

### Modeling of spore memory of germinant stimuli

First, we reproduce with our mathematical model an earlier published percentage germination curve showing spore memory for stimulation of wild-type *B. subtilis* spores with L-valine.^32^ Next, we study the relationship between the mean *E*(*R*_i_) of the GR distribution and the percentage germination upon the 1^st^ and the 2^nd^ germinant pulses. We then compare the predictions with experimental results of the percentage germination of wild-type spores and mutant spores with elevated GR levels. Wild-type spores can acquire memory upon short pulses of L-valine to induce germination as follows. After the 1^st^ pulse a fraction of the spores is committed to germinate and does so. From the subpopulation that does not germinate after the 1^st^ pulse, a fraction germinates upon a 2^nd^ pulse and finally a fraction does not germinate at all. Interestingly, spore populations can exhibit 2- to 3-fold more germination in the 2^nd^ germinant pulse following the 1^st^ one. Apparently, there is memory in the molecular system for external perturbations such as a germinant pulse that occurred before the 2^nd^ pulse.^32^ Consequently, we optimize model parameters to reproduce the spore germination curve for an experiment with two short germinant pulses.^32^ In our simulation, we select two pulses of germinant exposure from 0 < *t* < 5 min and 30 < *t* < 35 min. We set the germinant concentration to 3.5 mM. A percentage germination curve in our definition is a cumulative distribution function of *T*_open_ to give rapid CaDPA release. Spores that do not germinate have an infinite *T*_open_ and do not contribute to the distribution in the time frame of the simulations. Heterogeneity of *T*_open_ and as result *T*_lag_ determines the shape of the curve. Here, we introduce heterogeneity through a gamma distribution of GR copy numbers (Equation 2). For each simulation of a cycle of 500, we randomly select a GR copy number from a gamma distribution but keeping SpoVA copy numbers constant. This means that *C*_c_ + *C*_ca_ + *C*_o_ is constant in each cycle of a set of simulations. This approach is justified by the experimental observation that overexpression or deletion of GR’s has no effect on levels of SpoVAD and by inference levels of all proteins encoded by the heptacistronic *spoVA* operon.^15^ From western blotting, it is known that on average ∼700 to ∼1,100 of each GR are present in a spores’ germinosome. The starting value of at least SpoVAD is 6,500 as determined earlier by western blotting.^20^ By tuning of *k*_4_, the SpoVA copy number of activated and open channels in our simulations asymptotically approaches 6,500 copies/spore (*vide infra*). This copy number is also an arbitrary selection as we do not know by experimentation the number of open SpoVA channels at the end of rapid CaDPA release. At the end of the simulations, we construct a predicted percentage germination curve. The experimental germination curve was determined by monitoring germinating spores with DIC microscopy. However, most of the intensity change seen in DIC microscopy is caused by rapid CaDPA release, and the kinetic parameters for which averages were determined are *T*_lag_, *T*_release_, and Δ*T*_release_ = *T*_release_−*T*_lag_.^28^^,^^32^^,^^46^

**Figure 1:**
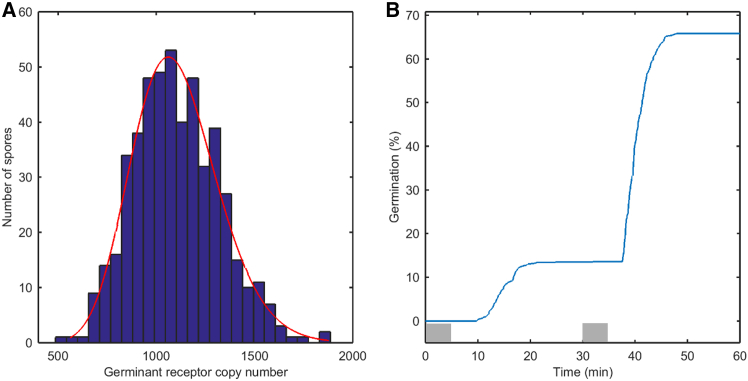
Simulation of spore germination memory (A) Gamma distribution of GR copy numbers used for simulation of the percentage germination efficiency curve (Figure 1B) with *E*(*R*_i_) = 1100 at *t* is 0 min. The red line in (A) is the fitted distribution. The GR estimated mean is 1,105 molecules/spore, and the estimated standard deviation is 219 molecules/spore; the parameters of the fitted gamma distribution are: *a* is 25.4491 [22.5002, 28.7844] and *b* is 43.4004 [38.3247, 49.1484]. (B) Percentage germination in a two pulse germination experiment. After the 1^st^ pulse the curve levels off to an asymptote at 15%. After the 2^nd^ pulse the curve approaches 68%. Two pulses of 3.5 mM germinant for receptor *R*_i_ activation are administered from 0 < t < 5 and 30 < t < 35 min. At zero time *R*_a_ = 0 copies/spore; *C*_c_ = 6,500 copies/spore. *C*_a_ = *C*_o_ = 0 copies/spore; *k*_1_ = 14e−3; *k*_-1_ = 100e−3; *k*_*2*_ = 25e−7; *k*_3_ = 60e−3; *k*_4_ = 195e2; *k*_5_ = 305e−2; *n* = 3; *θ* = 20.

**Figure 2:**
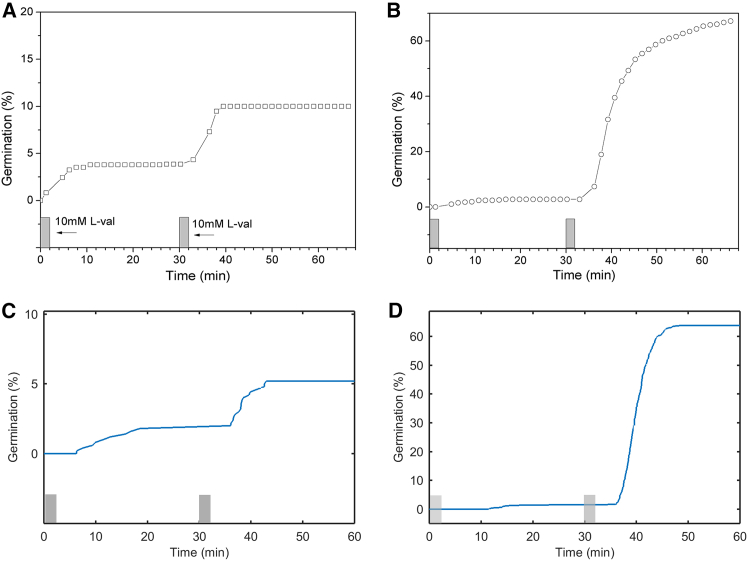
A double pulse germination experiment with *B. subtilis* wild-type spores and spores of an isogenic strain that overexpresses the GerA GR (A) Wild-type *B. subtilis* spores (PS533) were given two 2-min 10 mM L-valine (L-val) germinant pulses separated by 30 min. (B) Spores of *B. subtilis* PS3476 (GerA GR over-expressed ∼8-fold) were given two 2-min 10 mM L-valine germinant pulses separated by 30 min. Gray bars above the horizontal axis indicate pulse durations. (C) Simulated percentage germination of WT spores in a two pulse germination experiment. The estimated distribution mean is 1,118 and the standard deviation is 1,064 with *a* is 1.10476 [0.989585, 1.23334] and *b* is 1012.19 [881.615, 1162.1]. At zero time *R*_a_ = 0 copies/spore; *C*_c_ = 6,500 copies/spore. *C*_a_ = *C*_o_ = 0 copies/spore; *k*_1_ = 350e−5; *k*_-1_ = 100e−3; *k*_*2*_ = 25e−7; *k*_3_ = 60e−3; *k*_4_ = 195e2; *k*_5_ = 305e−2; *n* = 3; *θ* = 20. (D) Simulated percentage germination of overexpressed GerA GR spores in a two pulse germination experiment. The estimated distribution mean is 3,367 and the standard deviation is 399 with *a* is 71.2149 [62.9306, 80.5898] and *b* is 47.2855 [41.7667, 53.5336]. At zero time *R*_a_ = 0 copies/spore; *C*_c_ = 6,500 copies/spore. *C*_a_ = *C*_o_ = 0 copies/spore; *k*_1_ = 350e−5; *k*_-1_ = 100e−3; *k*_*2*_ = 25e−7; *k*_3_ = 60e−3; *k*_4_ = 195e2; *k*_5_ = 305e−2; *n* = 3; *θ* = 20.

We start with varying the mean *E*(*R*_i_) of the GR distribution and keep the standard deviation *Var*^½^(R) fixed. The parameters of the randomly accessed gamma distribution are estimated at the end of a set of 500 cycles from the fitted gamma distribution. A typical GR protein distribution is depicted in Figure 1A. The average of this GR copy number distribution is set to 1,100 copies/spore, and we selected combinations of distribution parameters *a* and *b* with the condition that *Var*^½^(x) = *a*^*½*^*b* = 220. With this setting the GR distribution tails to higher copy numbers, and a fraction of the spores germinate after the 1^st^ pulse and a fraction germinates after the 2^nd^ germinant pulse (Figures 1A and 1B). Notably, with the 1^st^ pulse 15% of the spore population germinates, while after the 2^nd^ pulse 68% of the remaining spores germinate. Although the germinant concentration and pulse lengths are equal for both pulses, germination after the 2^nd^ pulse is approximately 4-fold higher than after the 1^st^ pulse. Thus, the model reproduces published experimental data.^32^ Simulations for other *E*(*R*_i_) are compiled in Figure S1. We repeated a double pulse germination experiment with *B. subtilis* wild-type spores and spores of an isogenic strain that overexpresses the GerA GR ∼8-fold as determined by western blotting, with an experimental error of 25%.^7^ The experimental percentage germination curves are depicted in Figures 2A and 2B. We simulated the curves with our model (Figures 2C and 2D). Compared to germination of wild-type spores we had to adjust reaction rate constants for a 2 min pulse for wild-type spores that overexpress GerA with 10 mM L-valine as germinant. A broad GR copy-number distribution, with *E*(*R*_i_) at 1,100, *a* is 1 and *b* is 1,100, reproduces approximately 4% germination upon the first pulse and approximately 6% germination at the second pulse (Figure 2C). Next, we overexpress the GerA GR by increasing *E*(*R*_i_) and keep the reaction rate constants similar. In this approach, we put forward the constraint that overexpression of GR GerA from a more active promotor does not affect the rate constants for binding and dissociation of the germinant to the receptor. A second assumption is that expression of GerA from another promotor affects the parameters of the gamma distribution. It is known from modeling of burst like stochastic gene expression that the rate at which the gene transitions from the inactive state to the active state and vice versa determines the momenta of the mRNA copy number distribution and consequently the protein copy number distribution.^47^ Our modeling results are in accord with an earlier prediction using a stochastic gene expression model that a high burst frequency produces a narrow mRNA copy-number distribution and a low burst frequency a broad mRNA distribution.^47^ However, experimental data for the distribution of copy numbers of an overexpressed GFP-labeled GerA in individual spores are lacking. With *E*(*R*_i_) at 3,350 *a* as 67 and *b* as 50 we reproduce 3% germination at the first pulse and 64% germination at the second pulse (Figure 2D). In this case, we had to adjust the variance of the GR distribution to reproduce the percentage germination curve. For *E*(*R*_i_) at 3350 the overexpression is approximately 3.3-fold. For further increase of *E*(*R*_i_) we have to diminish *k*_1_ to keep the second pulse at 63% without surpassing the 3% germination at the first pulse. These results validate simulations of the relation between GR protein distributions and percentage germination curves in a double pulsed memory experiment. Interestingly, the model predicts constant Δ*T*_release_ times and that Δ*T*_release_ is independent of *T*_lag_, corroborating observations from earlier experimental work.^28^ Next, we probed for the effect of varying the concentration of germinants used during the pulses, from 2 mM to 5 mM (Figure S2). At 2 mM germinant no germination is predicted with the 1^st^ pulse and approximately 1% after the 2^nd^ pulse. This latter percentage increases to 12% at 2.5 mM germinant but still with no germination after the 1^st^ pulse. This implies that the 1^st^ pulse primes 12% of the spore population for germination after the 2^nd^ pulse. For higher germinant concentrations, significant germination with the 1^st^ pulse as well as substantial memory effects are predicted, e.g., Figure S2C. The influence of the standard deviation of the distribution is further highlighted in Figures S3 and S4. There is an obvious relationship between the variance of the distribution and the fraction of spores germinating after the 1^st^ and 2^nd^ pulses. As the variance of the distribution increases and tails to higher GR copy numbers, more spores germinate with the 1^st^ germinant pulse.

### Dynamics of SpoVA channel opening

**Figure 3:**
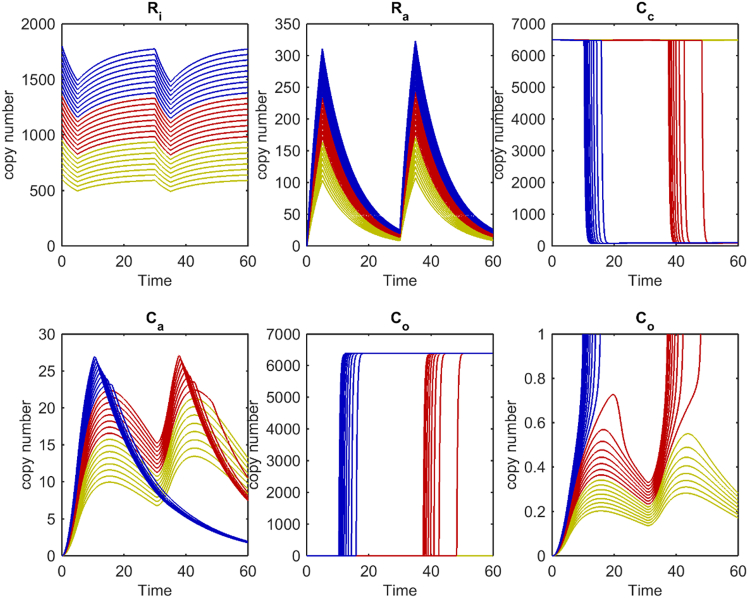
The copy numbers of R_i_, R_a_, C_c_, C_a_, C_o_, and an extension of C_o_ as a function of time Each trace represents a state variable profile for a variable GR *R*_i_ copy number. The starting GR values are 600 ≤ *R*_i_ ≤ 1800 copies/spore and the increment is Δ*R*_i_ = 50 copies/spore. The yellow spore population (600 ≤ *R*_i_ ≤ 950) does not germinate, the red one (1000 ≤ *R*_i_ ≤ 1350) germinates at the 2^nd^ pulse and the blue ones (1400 ≤ *R*_i_ ≤ 1600) germinate at the first pulse. Two pulses of 3.5 mM germinant for *R*_i_ activation are administered from 0 < t < 5 and 30 < t < 35 min. At time zero, *R*_a_ = 0 copies/spore; *C*_c_ = 6,500 copies/spore. *C*_a_ = *C*_o_ = 0 copies/spore; *k*_1_ = 14e−3; *k*_-1_ = 100e−3; *k*_*2*_ = 25e−7; *k*_3_ = 60e−3; *k*_4_ = 195e2; *k*_5_ = 305e−2; *n* = 3; *θ* = 20.

The kinetics of SpoVA channel opening are studied in more detail for GR copy numbers ranging from 600 to 1,800 molecules/spore. The increment of GRs is 50 copies per cycle of numeric evaluation of the set of ordinary differential equations (ODEs) in a simulation. The number of GRs is defined by *R*_i_ and at time point *t* is 0 s. The results *C*_o_ and the copy-number time profiles of the state variables *R*_i_, *R*_a_, *C*_c_, and *C*_ca_ are given in Figure 3. There are three subpopulations in the uniform GR distribution. With 600 to 950 GRs/spore, there is no germination, with 1,000–1,350 GRs/spore there is germination after the 2^nd^ pulse and with 1,400–1,800 GRs/spore there is germination after the 1^st^ and 2^nd^ pulses. Figure 3 shows that only in a fraction of the spore population are GRs sufficiently activated by germinant binding to lead to commitment to germinate. We can tune this fraction by variation of the rate constants *k*_1_ and *k*_*-*1_ for binding of *S* to GR and dissociation thereof. This tuning also affects the number of spores opening SpoVA channels during the 1^st^ and 2^nd^ pulses. With the parameter settings of our simulation only a few SpoVA channels are activated by interaction with GRs. Because of different copy numbers between GRs and channels there cannot be a one to one stoichiometry between GR activation and channel opening. Approximately 1,100 GerA GRs are in the germinosome, while ∼6,500 SpoVA channels are dispersed over the IM. The surface area of the spore IM can be approximated by an ellipsoid. The surface area of an ellipsoid (*A*_im_) is calculated as *A*_im_ = 2π(*s*^2^+*ls*), where *l* is the length of the long axis and *s* of the short axis.^48^ The long axis *l* and short axis *s* of the IM are estimated by electron microscopy and *l* is 837 nm and *s* is 554 nm for a typical spore.^49^ These are obtained by subtraction of coat and cortex layers’ thickness from the spore long axis and short axis.^49^ The surface area of the IM is *A*_im_ = ∼4,835,800 nm^2^, and on average there will be one SpoVA molecule per 744 nm^2^ of IM. Next, we calculate the area of the germinosome. As input, we use the normalized average distribution of the fluorescence signal along the long axis of a spores. The full width at half max intensity for GerKB-GFP signal is 300 nm^9^. Assuming a planar circle on the surface of the IM for the contour of the germinosome its surface *A*_ger_ = π*r*^2^ = 502,655 nm^2^. Assuming a homogeneous distribution of 744 nm^2^ per SpoVAE then the germinosome contains at most 676 SpoVA molecules. In a stoichiometry where one GR activates one channel maximally, 1,100 channels can be activated. Thus, there are ∼5,800 SpoVA channels that cannot directly interact with a GR. Thus, SpoVA channel cooperativity is introduced in the model to relay the signal from GRs over the entire IM surface to open more than approximately 676 channels. In our model, closed SpoVA channels are directly opened by activated and open SpoVA channels. Immediately after the 1^st^ germinant pulse the number of open SpoVA channels gradually increases. This slow increase is the gross difference between opening by activated SpoVA channels through *k*_4_*C*_a_ and closing of SpoVA channels through decay –*k*_5_*C*_o_ (Equation 7). Beyond 10 min sufficient channel opening takes place to force cooperative opening of the dark blue subpopulation through the Hill function term in Equation 5. Channel opening is autocatalytically driven, all SpoVA channels open, and hence the *C*_*o*_ curve approaches 6,500 molecules. The SpoVA channels from spores that germinate after the 2^nd^ pulse are partially activated by the 1^st^ pulse but remain below a critical copy number needed to force an autocatalytic opening mediated by the Hill function in Equation 6. However, the extent of channel opening does not fully return to the baseline. A 2^nd^ pulse at 30 < *t* < 35 min contributes additionally to channel opening and drives through cooperative action channel opening to completion at 6,500 copies/spore. As a result, channels open that are not opened by the 1^st^ germinant pulse. Thus, the system exhibits memory for an earlier germination stimulus. Finally, the curve levels off to a plateau at 6,500 open SpoVA channels. The level of the plateau of open SpoVA channels is mainly determined by *k*_4_ and *k*_5_. A third fraction does not open at all, as the 2^nd^ germinant pulse is insufficient to trigger autocatalytic SpoVA opening and the number of open channels decays to the baseline.

## Discussion

Spore germination experiments with sequential germinant stimuli separated in time are very suitable to construct and optimize a kinetic germination model. The shape of the percentage germination curves with multiple inflection points and asymptotes enables a more robust fitting of model parameters than a single stimulus sigma-shaped germination response experiment. The germination model based on the minimal molecular network as presented in Figure 1 and described by differential Equations 3, 4, 5, 6, and 7 reproduces percentage germination curves in double pulse memory experiments^32^ and newly acquired data (Figures 2A and 2B). Upon germinant exposure in two 5 min pulses separated by 30 min, two subpopulations of the whole population germinate. The second one exhibits a memory effect, in that the amounts of spores that germinate after the 2^nd^ pulse are larger than those germinating after the 1^st^ pulse. Memory has been attributed to a conformational change in some germination protein that activates this protein for germination, although the activated protein can also decay to the inactivate ground state. When the activated protein is not fully decayed, a 2^nd^ germinant pulse leads to a larger population of activated proteins and a higher percentage of germination after the 2^nd^ pulse.^32^ The reconstructed network shows that a minimal set of an inactive and active GR and a transport channel in three states—closed inactive, closed active, and open is needed to reproduce memory. Thus, memory can reside with GRs and/or be stored in the CaDPA channel complex. This is consistent with generation of memory in spores that lack all GRs when germination is triggered by direct or indirect stimulation of CaDPA release by exogenous CaDPA itself that activates CwlJ, or by dodecylamine that may cause the SpoVA channels to open.^32^

Memory is stored in an activated GR *R*_a_, although this can decay to the inactive state *R*_i_. As is seen in Figure 3, GR inactivation does not fully return to the baseline before the 2^nd^ germinant pulse and thus GR inactivation characteristics contribute to memory. The fraction of activated GRs and its relaxation to an inactive state is determined in our model by the ratio *k*_1_/*k*_-1_, although we do not know this ratio from experimental data. However, memory in our model is not uniquely confined to GR activation and inactivation kinetics, as there is a model parameter setting with *k*_-1_ = 225 × 10^−3^ and *k*_2_ = 45 × 10^−7^ where *R*_a_ returns to baseline before the 2^nd^ germinant pulse, and the network still shows memory for germination without adjusting the other rate constants. In this case memory is introduced in the network by the interplay between activation of closed channels and their rate of opening to *C*_o_. The spore channel activation rate is proportional to *k*_2_*R*_a_ and the number of closed inactive SpoVA channels *C*_c_ (Equation 6), and the rate of opening is proportional to *k*_3_*C*_a_. We assume that the number of activated GRs at 3.5 mM is approximately 17% and we have optimized *k*_2_ accordingly. This needs further experimentation to dissect *k*_2_*R*_a_ into *k*_2_ and *R*_a_. Rate constant *k*_3_ has an effect on *T*_lag_ because it feeds the rate of open channel formation (Equation 7). The Hill coefficient *n*, *θ* together with *k*_4_ and *k*_*5*_ determine the bistability of the model needed to irreversibly open CaDPA channels. In this simulation, *n* is 3 pointing to cooperativity effects of open activated SpoVA channels (Equation 7). Furthermore, rate constants *k*_4_ and *k*_5_ in combination with *θ* determine the number of open SpoVA channels at the endpoint of simulations. This number is not known by experimentation and is arbitrarily set.

The time to the onset of rapid CaDPA release *T*_lag_ is heterogeneous and shows a probability distribution. We introduce germination time heterogeneity through the GR distribution in a spore population. In a cycle of computer simulations, we keep the rate constants fixed. Rate constants *k*_1_ and *k*_-1_ are on-rate and off-rate constants and determined by the 3D structure of the GR proteins. As the primary sequence of the GR proteins is not variable within an isogenic strain, we expect that a *k*_1_ and *k*_-1_ probability distribution does not contribute to heterogeneity of *T*_lag_ at the population level. At present, our model is a minimal one, and we do not differentiate between actions of GerA, GerB, and GerK GRs. Consequently, we do not integrate the signal from different GRs and germinants. We also do not know the fraction of SpoVA channels in an inactive state that is activated by a population of activated GRs. The network in our model is capable of signal amplification, as with current parameter settings approximately 1–27 closed SpoVA channels are activated (Figure 3). This is sufficient to irreversibly open the entire channel population through cooperative opening which is needed to open channels outside the perimeter of the germinosome. Another benefit from the network wherein only fractions of molecular populations are active is that it dampens fluctuations in germinant exposure. This ensures that there is a threshold for GR activation that will prevent unwanted early germination.

The model generated in this work has been optimized to reproduce memory of germinant exposures. Other combinations of reaction rate constants will likely also give kinetics that show memory, but the molecular wiring and the kinetic differential equations will likely remain identical. However, further, experimentation is needed to narrow down the parameter space. In particular, the contributions of GR activation and deactivation, and SpoVA channel activation, deactivation, and opening should be studied. The endpoint of germination in our model is the rapid opening of SpoVA channels. In a future extension of the model, the effects of the CLEs SleB and CwlJ should also be included. In addition, as noted previously GR-independent germination by CaDPA or dodecylamine also show memory, presumably through effects on the SpoVA channel alone, and this can also be introduced into the model. The model in its current state will be used in future research to reduce the number of experimental variables in an iterative cycle of prediction of the dynamics of a hypothetical molecular network, the design of an experiment, comparison with the outcome, and further tuning of the model until agreement between theoretical predictions and experimental data is reached.

While the mathematical model in this work is general, Equations 3, 4, 5, 6, and 7 can be simplified and lead to Woese et al.’s model^35^ and Li et al.’s model^36^ with specific values of parameters, while giving the biochemical insight of the trigger signal or germination substance P. Equation 4 can be rewritten as

(Equation 8) $$\frac{dR_{a}}{\text{dt}}=k_{1}S\left(R_{0}-R_{a}\right)-k_{-1}R_{a}\text{,}$$

where R_0_ = R_i_+R_a_ is the total GR copies/spore at t = 0. The solution of Equation 8 is given by

(Equation 9) $$R_{a}=\frac{k_{1}S}{k_{-1}{+k}_{1}S}\left[1-e^{-\left(k_{-1}+K_{1}S\right)t}\right]\text{,}$$

where the steady-state occupation of the active GR number is ${\bar{R}}_{a}=\frac{k_{1}S}{k_{-1}{+k}_{1}S}\;R_{0}$. The characteristic time for R_a_ reaches its steady-state value as *t*_GR_ = 1/(*k*_*1*_S + k_*-1*_). It should be noted that the value of k_1_ estimated for the simulation in Figures 2 and 3 gives relatively low occupation of the active GRs (25.9%–32.8%), far from saturation occupation. Because of the high number of GRs (1,100–3,300 copies/spore), there are still enough GRs being active to produce memory.

Equations 5, 6, and 7 can be written down for *k*_4_ = 0 and very large *k*_3_ value as

(Equation 10) $$\frac{dC_{o}}{dt}=\left(k_{2}C_{tot}\right)R_{a}-\left(k_{5}+k_{2}R_{a}\right)C_{o}\text{,}$$

where *C*_*tot*_ is the total SpoVA channel number (∼6,500 copies/spore). For the situation, *t* ≫ *t*_GR_, the commitment time is much longer than the characteristic time for GR activation by germinant binding, the solution of Equation 10 is given by

(Equation 11) $$C_{o}\left(t\right)=\left(\frac{k_{2}R_{a}}{\beta }\right)C_{tot}\left(1-e^{-\beta t}\right)\text{,}$$

where *β=k*_*5*_*+k*_*2*_*R*_*a*_. Equations 8 and 10 are basic equations used in Woese et al.’s work^35^ and Li et al.’s work,^36^ which can well explain germination heterogeneity, commitment, and memory. When *C*_*o*_ level/spore reaches a threshold (50 copies used in this work), the spore commits to germinate by releasing its core’s CaDPA. Obviously, the trigger signal or germination substance P is the number *C*_*o*_ of SpoVA channels per spore in the open and activated state, and memory appears in sub-threshold open SpoVA channel number *C*_*o*_. From Equation 11, due to the long-decaying parameter β, the sub-threshold channel number *C*_*o*_ will not immediately decay to zero after the first germinant pulse, and it can be accumulated to a higher level until the germination threshold when the second germinant pulse arrives to activate the GRs. Accordingly, the key parameters that influence the model predictions are *k*_5_ and *k*_2_*R*_*a*_.

### Limitations of the study

Although this mathematical model successfully predicts memory, its validation relies on a deeper understanding of the structure and function of GRs and SpoVA channel proteins. First, while the GerAA, GerAB, and GerAC subunits have been identified for detecting germinants like L-alanine or L-valine and activating GerA receptors, the rate constants for germinant binding and unbinding to the GerAB subunit remain unclear. Additionally, the mechanism by which bound GerAB subunits activate GerA receptors is not well understood. Thus, the rate constants *k*_1_ and *k*_-1_ depend on a comprehensive understanding of GRs. Second, the precise distribution of GRs in a given spore preparation, modeled as a Gamma distribution in this work, depends on the experimental determination of GR protein levels. Third, the mechanisms by which activated GRs transfer signals to activate SpoVA channel proteins and the duration of SpoVA channel activation remain unclear, which impacts the values of *k*_2_ and *k*_5_. Finally, the mechanism behind the germination threshold is not fully understood, which effects the selection of the threshold level (50 copies used in this work).

### Computational modeling and simulations

Simulations were performed in MATLAB (MathWorks, Natick, MA, USA). Custom MATLAB scripts are provided in supplemental information MATLAB Data S1 and S2. The MATLAB scripts were developed to simulate.

• Percentage germination curves.
• Germinant receptor activation dynamics
• SpoVA channel state transitions

### Model description and parameters

The stochastic model describes GR activation and SpoVA channel gating. At *t* = 0, set *R*_*a*_ = 0, *C*_*ca*_ = 0, and *C*_*o*_ = 0. Inactive GR molecules (*R*_*i*_) follow a gamma distribution with a mean of ∼1,100 molecules per spore. Closed SpoVA channels (*C*_*c*_) are present at ∼6500 copies per spore. SpoVA channel gating cooperativity is described by a Hill coefficient (n) and dissociation constant (θ), where θ^n^ defines half-maximal *C*_*o*_ production. Simulations were carried out using the MATLAB package of software (the MathWorks, Natick, USA). M-files with source code for simulation of percentage germination curves and copy number time profile plots are given in supplemental information Data S1 and S2. The model parameters are listed in the table below.

**Table:** 

Table of model parameters	Parameter description
*R*_*i*_	number of inactive GR molecules/spore. The levels of *R*_*i*_ in a spore population in the absence of germinant vary according to a gamma distribution function. The estimated mean of *R*_*i*_ level is ∼1100 molecules/spore.
*R*_*a*_	number of active GR molecules/spore. The level of *R*_*a*_ = 0 copies/spore at *t* = 0 or in the absence of germinant.
*C*_*c*_	number of SpoVA channel proteins/spore in the closed state. The estimated level of *C*_*c*_ ∼6500 copies/spore in the absence of germinant.
*C*_*ca*_	number of SpoVA channel proteins/spore in the activated closed state. *C*_*ca*_ = 0 copies/spore at *t* = 0
*C*_*o*_	number of SpoVA channel proteins/spore in the open state. *C*_*o*_ = 0 copies/spore at *t* = 0.
*k*_1_	the rate constant of converting an *R*_*i*_ GR to *R*_*a*_ by germinant *S* binding. *k*_1_*S* is the rate of converting an *R*_*i*_ GR to *R*_*a*_ GR.
*k*_-1_	the rate constant for a *R*_*a*_ GR decaying to *R*_*i*_ due to the reversible dissociation.
*k*_2_	the constant rate of converting a closed SpoVA channel *C*_*c*_ to an activated closed channel *C*_*ca*_ by active GR *R*_*a*_. *k*_2_*R*_*a*_ is the rate of converting an *C*_*c*_ channel to *C*_*ca*_ channel.
*k*_3_	the rate constant of converting an activated closed channel *C*_*ca*_ to an open channel *C*_*o*_.
*k*_4_	the maximum production rate of open SpoVA channel *C*_*o*_ due to non-linear bistability.
*k*_5_	the rate constant for an open channel *C*_*o*_ decaying to a closed SpoVA channel *C*_*c*_.
*S*	germinant concentration, which binds to GR molecules.
*n*	Hill coefficient, describing Cooperativity of SpoVA channel gating.
*θ*	dissociation constant. *θ*^*n*^ sets the value at which the production rate of *C*_*o*_ is half its maximum value.

## Resource availability

### Lead contact

• Requests for further information and resources should be directed to and will be fulfilled by the lead contact, Stanley Brul (s.brul@uva.nl).

### Materials availability

• This study did not generate new unique reagents.

### Data and code availability

• Data reported in this paper will be shared by the lead contact upon request.
• All MATLAB scripts used for simulation of germination kinetics and SpoVA channel dynamics are available in supplemental information (supplemental MATLAB Data S1 and Data S2).
• Additional information is available from the lead contact upon reasonable request.

## Acknowledgments

C.G.d.K., L.J.d.K., and S.B. acknowledge the 10.13039/501100001827University of Amsterdam for funding of their research. In memory of prof. Chris de Koster who passed away 5th August 2019.

## Author contributions

C.G.d.K. developed the model and performed computational analyses with input from L.J.d.K. and S.B.; X.L. and Y.Q.L. conducted experiments; P.S. provided spores of *Bacillus subtilis* strains; C.G.d.K. wrote the manuscript with input from L.J.d.K., S.B., Y.Q.L., and P.S.; Y.Q.L., X.L., and P.S. designed the germination experiments.

## Declaration of interests

The authors declare no competing interests.

## STAR★Methods

### Key resources table

**Table:** 

REAGENT or RESOURCE	SOURCE	IDENTIFIER
**Bacterial strains**		

*Bacillus subtilis* PS533 (wild type)	Laboratory stock	Setlow lab. strain PS533
*Bacillus subtilis* PS3476 (GerA overexpression; ∼8× GerA)	Laboratory stock	Setlow lab. strain PS3476

**Chemicals**		

L-Valine (≥99%)	Thermo Scientific Chemicals (Thermo Fisher Scientific)	Cat A18894.06
HEPES	Sigma-Aldrich (Merck)	Cat 7365-45-9
Potassium hydroxide (KOH)	Sigma-Aldrich (Merck)	Cat 1310-58-3
Ultrapure water (Milli-Q grade)	Millipore	Milli-Q system

**Deposited data**		

MATLAB simulation scripts	This study	Supplemental MATLAB m-file one and two

**Software and algorithms**		

MATLAB	MathWorks	https://www.mathworks.com
Custom MATLAB simulation code	This study	Supplemental files

**Other**		

22 × 22 mm coverslips (#1 thickness, 0.13–0.16 mm)	Fisher Scientific	Cat 50-143-780
Nikon Ti inverted microscope	Nikon Instruments	ECLIPSE Ti Series
Plan Apo 60× oil objective (NA 1.4)	Nikon Instruments	Plan Apo 60× NA1.4
CCD camera (1392 × 1040 pixels; 12-bit)	Mightex Systems	CCE-B013-U
Custom autofocus system (650 nm diode laser + piezo actuator)	Custom-built	Piezosysrem Jena, MIPOS 100 PL

### Experimental model and study participant details

#### Bacterial strains and spore preparation

*Bacillus subtilis* strains PS533 (wild type) and PS3476 (GerA overexpression, ∼8-fold increased GerA levels) were used in this study. Spores were prepared, purified, and stored using standard procedures established in the Brul and Setlow laboratories. Briefly, spores were harvested from sporulating cultures, purified by repeated washing in sterile distilled water, and stored at 4°C in water until use. Spore preparations were >98% phase bright as determined by phase-contrast microscopy, indicating minimal contamination with vegetative cells, sporulating cells, or germinated spores. Prior to germination experiments, spores were heat activated at 70°C for 30 min in sterile water, followed by rapid cooling on ice for at least 15 min.

### Method details

#### Phase contrast microscopy

Spores (1 μL; ∼10^8^ spores/ml in water) were spread on the surface of a microscope coverslip (Fisher Scientific, 22 × 22 mm, #1 (0.13–0.16 mm)) that was then dried in a vacuum desiccator for 5 min. Coverslips were suspended in water and then mounted on and sealed to a microscope sample holder kept at a constant temperature of 37°C. A phase contrast microscope (Nikon Ti, equipped with an objective of Plan Apo 60×, NA1.4) was used to record the images of ∼500 individual spores at a rate of 15 s per frame for 60–90 min. Spores that contain DPA appear as bright and spores that do not contain DPA appear as dark. The percentage of spores containing DPA was defined as the number of bright spores divided by the total number of spores. A digital charge-coupled-device (CCD) camera (1,392 × 1,040 pixels and 12 bits) was used to capture the phase-contrast images. A home-made auto-focus system was developed to actively lock in the focus of the objective by using a diode laser at 650 nm to detect the distance change between the objective and the surface of the sample coverslip, which was fed back to a piezo actuator (Piezosystem Jena, MIPOS 100 PL) attached on the objective to lock the focus position.^30^^,^^33^

#### Spore germination with germinant pulses

*B. subtilis* spores of strains PS533 (wild-type) and PS3476 (overexpresses the GerA GR ∼8-fold) were prepared, purified and stored as described previously.^14^ The purified spores were free (>98%) of sporulating or growing cells or germinated spores as observed by phase contrast microscopy. *B. subtilis* spores were heat activated prior to nutrient germination by incubation in water at 70°C for 30 min and then by cooling on ice for at least 15 min. *B. subtilis* spores attached on the surface of a glass coverslip were germinated in 25 mM K-HEPES buffer (pH 7.4) with a pulse of a germinant (10 mM L-valine, Thermo Scientific Chemicals) for 2 or 4 min before the germinant was removed and spores were rinsed 5 times with 25 mM HEPES buffer (pH 7.4) using vacuum pump suction.^32^ The rinsed spores were then incubated at 37°C in 25 mM K-HEPES buffer with a pH of 7.4 unless noted otherwise. After various incubation times, the spores were given a 2^nd^ exposure to germinants for various times at 37°C in 25 mM K-HEPES buffer (pH 7.4) followed by germinant removal, rinsing by vacuum pump suction, and further incubation at 37°C in 25 mM K-HEPES buffer (pH 7.4).

### Quantification and statistical analysis

Images of ∼500 individual spores were analyzed in ImageJ. The percentage of germination from phase bright to phase dark spores was assessed with the aid of the ImageJ plugin https://sils.fnwi.uva.nl/bcb/objectj/examples/sporetrackerx/MD/sporetrackerx.html (custom made by Norbert Vischer). Quantitative analysis of germination memory was done as described previously.^32^ Further details are reported in figure legends.

## Supplemental information

Document S1. Figures S1–S4, Code Data S1 and S2
