# Supplementary material for: A mathematical kinetic model of memory in Bacillus subtilis spore germination
Source: iScience. 2026 Apr 22;29(6):115864. doi: 10.1016/j.isci.2026.115864 (PMC13186041; doi:10.1016/j.isci.2026.115864)
Supplement: Document S1. Figures S1–S4, Code Data S1 and S2 [file mmc1.pdf]

iScience, Volume *29*

Supplemental information

A mathematical kinetic model of memory

in *Bacillus subtilis* spore germination

Chris G. de Koster, Xiuping Lin, Yong-qing Li, Stanley Brul, Leo J. de Koning, and Peter Setlow

SUPPLEMENTAL INFORMATION

Supplementary Figure S1-S4.

Figure S1. The effect of the GR gamma distribution mean $E(R_i)$ at t is 0 min on percentage germination in the 1st and 2nd germinant pulses. The scale and rate parameters a and b are varied where the standard deviation $Var^{1/2}(R_i)$ at t is 0 min is 220 and the mean GR copy number $E(R_i)$ is (a) 900, (b) 1100 or (c) 1300. Two pulses of 3.5 mM germinant for receptor R_i activation are administered from $0 < t < 5$ and $30 < t < 35$ min. At zero time $R_a = 0$ copies/spore; $C_c = 6500$ copies/spore. $C_a = C_o = 0$ copies/spore; $k_1 = 14e\text{-}3$; $k_{-1} = 100e\text{-}3$; $k_2 = 25e\text{-}7$; $k_3 = 60e\text{-}3$; $k_4 = 195e2$; $k_5 = 305e\text{-}2$; $n = 3$; $\theta = 20$.

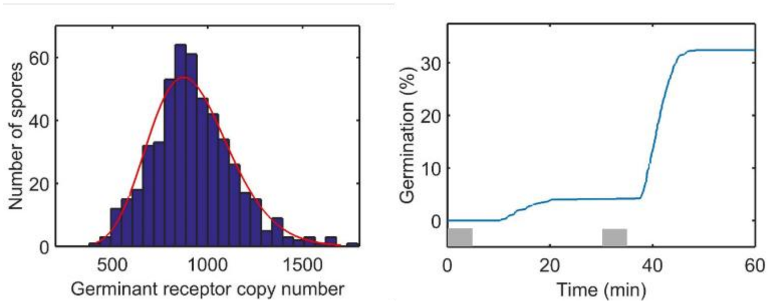

Figure S1(a). Left Panel: Gamma distribution of GR copy numbers (Left panel) used for simulation of the percentage germination efficiency curve (Right panel) with $E(R_i)$ is 900 at t is 0 min. The red line is the fitted distribution. The GR estimated mean is 924 molecules/spore, and the estimated standard deviation is 215 molecules/spore; the parameters of the fitted gamma distribution are: a is 18.489 [16.3515, 20.9059] and b is 49.9745 [44.1228, 56.6024]; Right panel: Percentage germination of spores in a two pulse germination experiment.

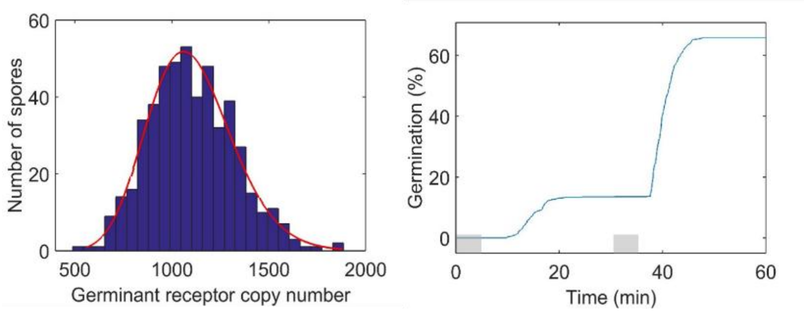

Figure S1(b). Gamma distribution of GR copy numbers (Left panel) used for simulation of the percentage germination efficiency curve (Right panel) with $E(R_i) = 1100$ at t is 0 min. The red line is the fitted distribution. The GR estimated mean is 1105 molecules/spore, and the estimated standard deviation is 219 molecules/spore; the parameters of the fitted gamma distribution are: a is 25.4491 [22.5002, 28.7844] and b is 43.4004 [38.3247, 49.1484]; Right panel: Percentage germination of spores in a two pulse germination experiment.

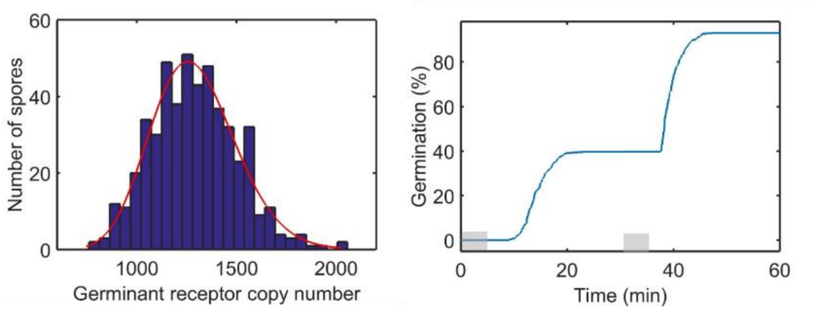

Figure S1(c). Left panel: Gamma distribution of GR copy numbers (Left panel) used for simulation of the percentage germination efficiency curve (Right panel) with $E(R_i) = 1300$ at t is 0 min. The red line is the fitted distribution. The GR estimated mean is 1292 molecules/spore, GR estimated standard deviation is 213 molecules/spore; the parameters of the fitted gamma distribution are: a is 36.8424 [32.5653, 41.6813] and b is 35.0556 [30.9598, 39.6931]; Right panel: Percentage germination of spores in a two-pulse germination experiment.

Figure S2. The dependence of percent germination of spores on germinant concentration *S*. Germinant *S* is varied from 2.0 mM to 5.0 mM with increments of 0.5 mM. The parameters and initial values of remaining variables at time zero are R_a = 0 copies/spore; C_c = 6500 copies/spore. $C_a = C_o$ = 0 copies/spore; k_1 = 14e-3; k_{-1} = 100e-3; k_2 = 25e-7; k_3 = 60e-3; k_4 = 195e2; k_5 = 305e-2; n = 3; θ = 20. The germinant receptor protein copy numbers are randomly drawn from a Gamma distribution with a = 25, b = 44 with $E(R_i)$ = 1100 and $Var^{1/2}(R_i)$ = 220 at t is 0 min.

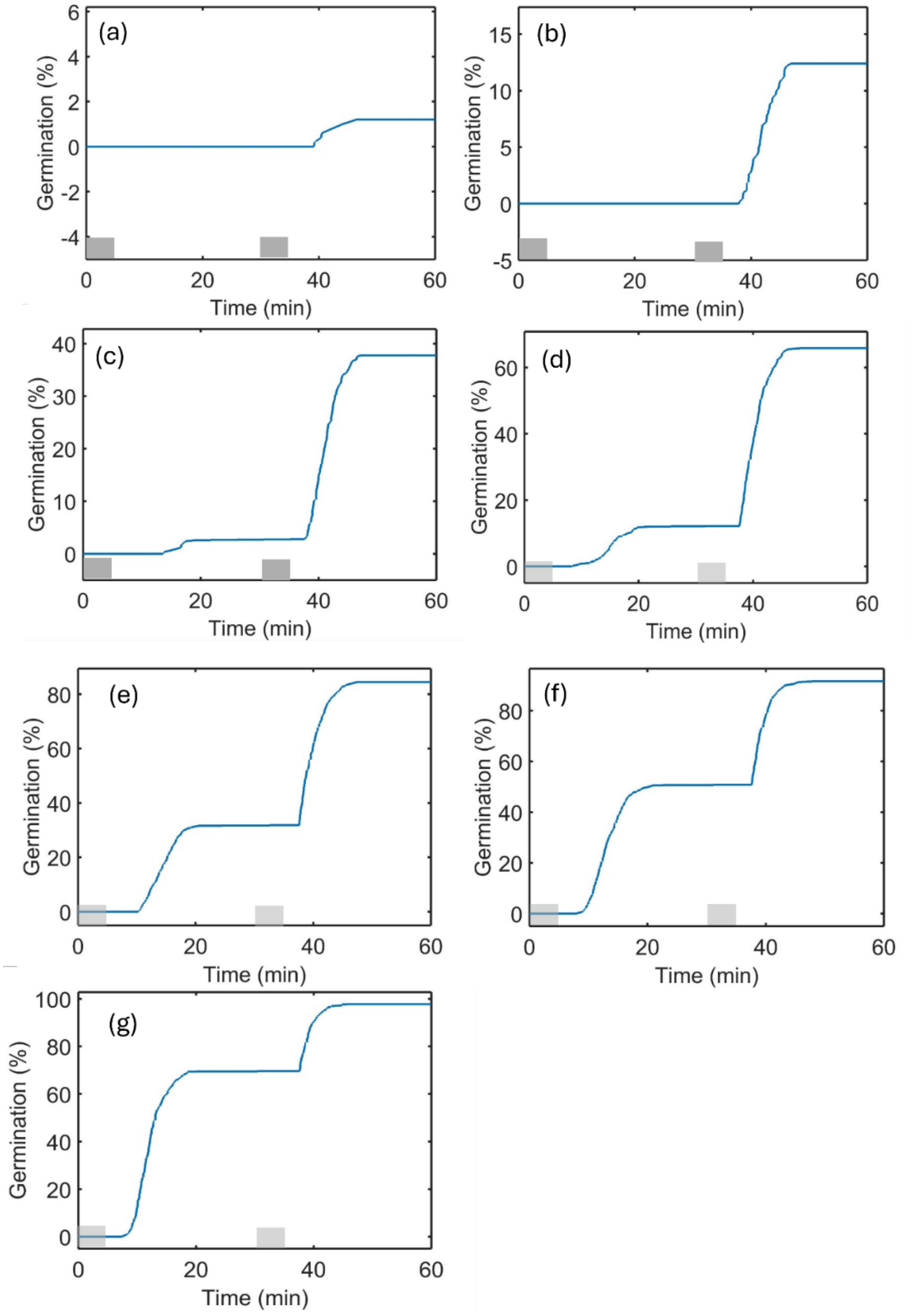

Figure S2(a-g). Percentage germination of spores in a two pulse germination experiment at (a) 2.0 mM, (b) 2.5 mM, (c) 3.0 mM, (d) 3.5 mM, € 4.0, (f) 4.5 mM, and (g) 5.0 mM germinant.

Figure S3. The influence of GR gamma distribution standard deviation on percent germination in the 1st and the 2nd germinant pulses. The scale and rate parameters *a* and *b* are varied where at *t* is 0 min the mean GR copy number is $E(R_i)$ = 1100 copies/spore and the standard deviation $Var^{½}(R_i)$ is 47 and 105 copies/spore. The parameters and initial values of remaining variables at time zero are R_a = 0 copies/spore; C_c = 6500 copies/spore. C_a = C_o = 0 copy/spore; k_1 = 14e-3; k_{-1} = 100e-3; k_2 = 25e-7; k_3 = 60e-3; k_4 = 195e2; k_5 = 305e-2; n = 3; θ = 20. The germinant receptor protein copy numbers are randomly drawn from a Gamma distribution.

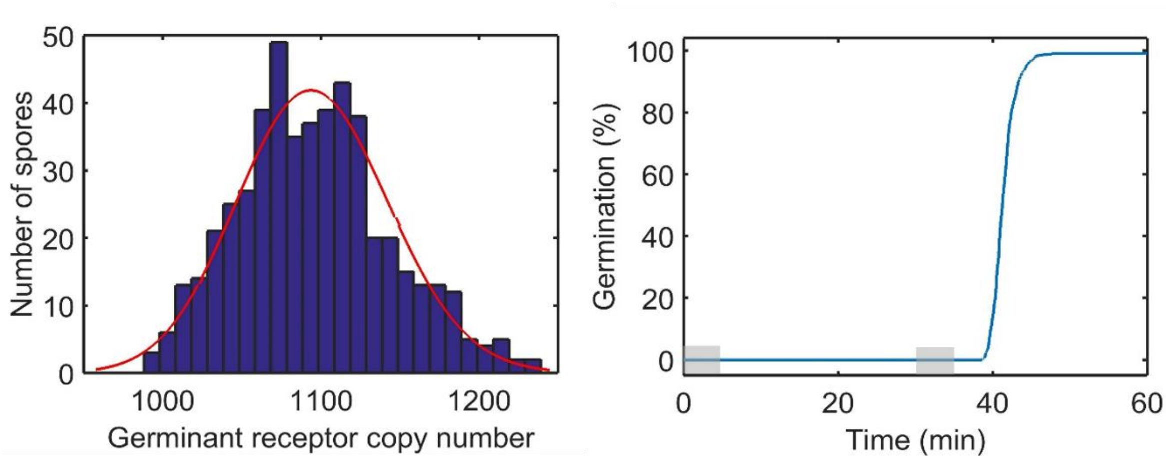

Figure S3(a). Left Panel: Gamma distribution of GR copy numbers used for simulation of the percentage germination efficiency curve (Right panel). The red line is the fitted distribution. $Var^{1/2}(R_{\mathrm{i}})$ is 47 at t is 0 min. The GR estimated mean is 1096 copies/spore, and the GR estimated standard deviation is 48 copies/spore; the parameters of the fitted gamma distribution are: a is 524.484 [463.356, 593.676] and b is 2.08941 [1.84578, 2.36519]. Right panel: Percentage germination of spores in a two-pulse germination experiment.

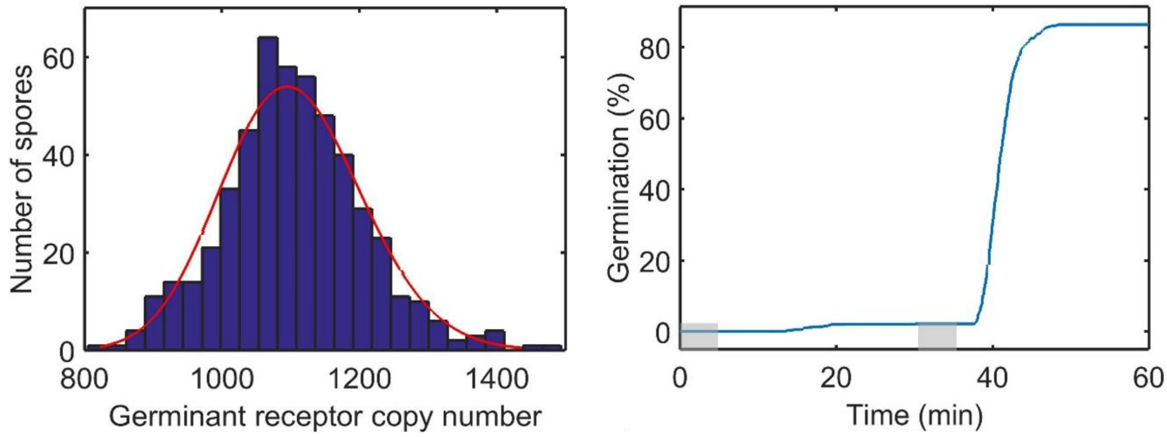

Figure S3(b). Left panel: Gamma distribution of GR copy numbers used for simulation of the percentage germination efficiency curve (Right panel). $Var^{1/2}(R_{\mathrm{i}})$ is 105 at t is 0 min. The red line is the fitted distribution. The GR estimated mean is 1105 copies/spore, and the GR estimated standard deviation is 102 copies/spore; the parameters of the fitted gamma distribution are: a is 116.917 [103.304, 132.323] and b = 9.44706 [8.34495, 10.6947]. Right panel: Percentage germination of spores in a two-pulse germination experiment.

Figure S4. The influence of GR gamma distribution standard deviation on percent germination in the 1st and the 2nd germinant pulses. The scale and rate parameters a and b are varied where at t is 0 min the mean GR copy number is $E(R_{\mathrm{i}})$ = 1100 copies/spore and the standard deviation $Var^{½}(R_{\mathrm{i}})$ is 348, 778 and 1100 copies/spore. The parameters and initial values of remaining variables at time zero are $R_{\mathrm{a}} = 0$ copies/spore; $C_{\mathrm{c}} = 6500$ copies/spore. $C_{\mathrm{a}} = C_{\mathrm{o}} = 0$ copy/spore; k_1 = 14e-3; k_{-1} = 100e-3; k_2 = 25e-7; k_3 = 60e-3; k_4 = 195e2; k_5 = 305e-2; $n = 3$; $\theta = 20$. The germinant receptor protein copy numbers are randomly drawn from a Gamma distribution.

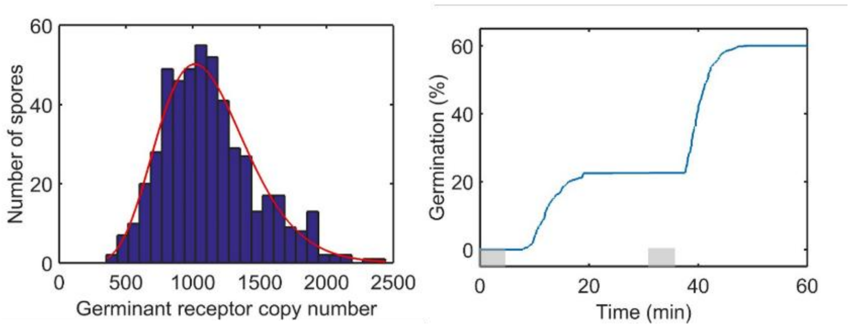

Figure S4(a). Left panel: Gamma distribution of GR copy numbers used for simulation of the germination efficiency curve (Right panel). $Var^{1/2}(R_{i})$ is 348 at *t* is 0 min. The red line is the fitted distribution. The GR estimated mean is 1122 copies/spore, and the GR estimated standard deviation is 348 copies/spore; the parameters of the fitted gamma distribution are: *a* is 10.4978 [9.29177, 11.8604] and *b* is 106.831 [94.2775, 121.055]. Right panel: Percentage germination of spores in a two pulse germination experiment.

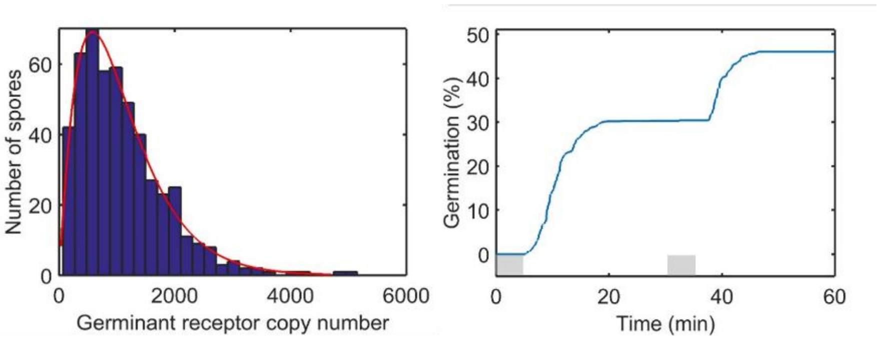

Figure S4(b). Left panel: Gamma distribution of GR copy numbers used for simulation of the percentage germination efficiency curve (Right panel). $Var^{1/2}(R_{i})$ is 778 at t is 0 min. The red line is the fitted distribution. The GR estimated mean is 1094 copies/spore, and the GR estimated standard deviation is 776 copies/spore; the parameters of the fitted gamma distribution are: *a* is 2.12876 [1.89643, 2.38955] and *b* is 513.649 [450.921, 585.103]. Right panel: Percentage germination of spores in a two-pulse germination experiment.

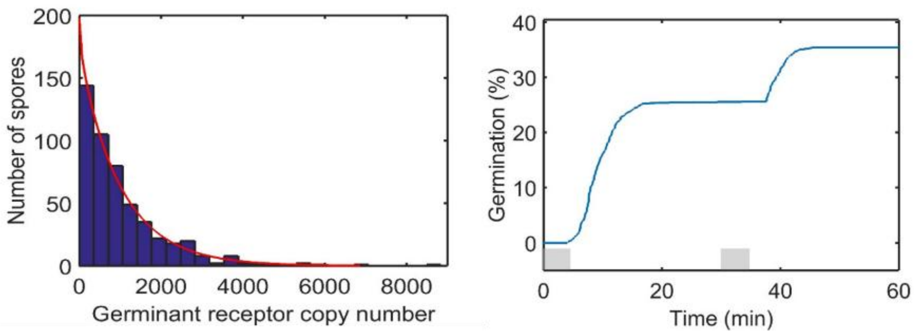

Figure S4(c). Left Panel: Gamma distribution of GR copy numbers used for simulation of the percentage germination efficiency curve. $Var^{1/2}(R_{i})$ is 1100 at t is 0 min. The red line is the fitted distribution. The estimated GR mean is 1023 copies/spore, and the estimated GR standard deviation is 1036 copies/spore; the parameters of the fitted gamma distribution are: *a* is 0.973473 [0.873043, 1.08546] and *b* is 1050.47 [912.749, 1208.96]. Right panel: Percentage germination of spores in a two-pulse germination experiment.

Supplementary Code DATA S1 and S2

<u>DATA S1.</u> This Matlab program simulates double-pulse spore germination dynamics by generating distributions of germinant receptor protein copy numbers, tracking open and active SpoVA channel states over time, and producing corresponding spore germination curves.

```
function [germinationtimes, pd, m, s] = percentagesporegermination

% The program generates a germinant receptor protein copy number
% distribution, open and active SpoVA copy number/spore time profiles and
% a percentage spore germination curve for a double pulse spore germination
% simulation.

clear all

Nsim = 500;       % number of simulations
Time = 60;        % time of simulation

figure('OuterPosition',[100 100 800 800]);

EE = { };
I = { };
T = { };

for j = 1 : 1 : Nsim

    a = 1;                                                      % parameter
gamma distribution
    b = 1100;                                                   % parameter
gamma distribution
    rec(j) = random('Gamma',a, b);                              % random
generator gamma distribution

   i   = 10.00001;                                              % germinant
concentration in mM
    tspan = [0 : 0.01 : Time];


    options = odeset('MaxStep', 0.005);
    y0 = [rec(j) 0 6500 0 0];                                   % defines
starting values of state variables
    protein = ode23(@kinetics, tspan, y0, options);

    t = protein.x;
    A = protein.y(1,:);
    B = protein.y(2,:);
    C = protein.y(3,:);
    D = protein.y(4,:);
    E = protein.y(5,:);                                         % open and
active SpoVA

    EE{1,j} = E;

    I{1,j} = find(EE{1,j} > 50, 1);
    T{1,j} = 0.005 * I{1,j};  %scale time by MaxStep
    numericVector = cell2mat(T);
    germinationtimes = sort(numericVector);
```

```


 hold on

   plot(t,E,'Color', '[0 0 0.75]');
   xlabel('Time (min)');
   ylabel('Copy number SpoVA open');
   fig.PaperUnits = 'inches';
    fig.PaperPosition = [0 0 5 5];
    fig.PaperPositionMode = 'manual';

end

   pd = fitdist(rec(:),'Gamma');
   m = mean(pd);
   s = std(pd);

figure;
histfit(rec(:),25,'Gamma');
xlabel('Germinant receptor copy number');
ylabel('Number of spores');


NGerminations = length(germinationtimes);
NGerminationsMin = min(germinationtimes);
NGerminationsMax = max(germinationtimes);

for l = 1 : 1 : NGerminations

    GG(l) = l;
end

figure;
plot([0 NGerminationsMin-0.1 germinationtimes NGerminationsMax+0.1 Time],
[0 0 100*GG/Nsim 100*NGerminations/Nsim 100*NGerminations/Nsim]);
axis([0 Time -5 100*NGerminations/Nsim + 5]);
xlabel('Time (min)');
ylabel('Germination (%)');

function dydt = kinetics(t,y)

if t < 2                  % defines timing of block pulses
signal = i;
 elseif t < 30
 signal = 0;
elseif t < 32
 signal = i;
else
    signal = 0;
 end

dydt = zeros(5,1);

% rate constants

k1  = 355e-5;
kr1 = 100e-3;
k2  = 25e-7;
```

```
k4 = 19500;
k3 = 60e-3;
k5 = 305e-2;

n = 3;
theta = 20;
synthesis = k4*y(5).^n ./ (y(5).^n + theta.^n);
decay =k5* y(5);

% differential equations

dydt(1) = -k1*signal*y(1) + kr1*y(2);            %Ger receptor
dydt(2) =  k1*signal*y(1) - kr1*y(2);            %Ger + germinant
dydt(3) = -k2*y(2)*y(3) + decay - synthesis;     %closed channel
dydt(4) =  k2*y(2)*y(3) - k3*y(4);               %closed channel activated
dydt(5) =  k3*y(4) + synthesis - decay;          %open channel


end
end
```

DATA S2. This Matlab program simulates the dynamic states of germination machinery by generating time-resolved copy number profiles of inactive and activated germinant receptors, as well as inactive closed, activated closed, and activated open SpoVA channel states at the single-spore level

```
function germinosomeproteintimeprofiles

% The program generates inactive and activated germinant receptor protein,
% inactive closed, activated closed and
% activated open SpoVA copynumber/spore time profiles.

clear all

figure('OuterPosition',[200 200 1400 700]);

for j = 600 : 50 : 1000                                 % starting value
inactive germinant receptor protein

   i  = 3.500001;                                       % germinant
concentration in mM
    tspan = [0, 60];

    options = odeset('MaxStep', 0.01);
    y0 = [j 0 6500 0 0];                                % defines starting
values of state variables {copy number/spore]
    protein = ode23(@kinetics, tspan, y0, options);

    t = protein.x;
    A = protein.y(1,:);                                 % inactive
germinant receptor protein
    B = protein.y(2,:);                                 % activated
germinant receptor protein
    C = protein.y(3,:);                                 % closed and
inactive SpoVA
    D = protein.y(4,:);                                 % closed and
activated SpoVA
    E = protein.y(5,:);                                 % open and
activated SpoVA

 hold on

   subplot(2,3,1);
   plot(t, A,'Color', '[0.75 0.75 0]');

   hold on

    subplot(2,3,2);
    plot(t, B, 'Color', '[0.75 0.75 0]');

    hold on

    subplot(2,3,3);
    plot(t, C, 'Color', '[0.75 0.75 0]');

    hold on

    subplot(2,3,4);
    plot(t, D, 'Color', '[0.75 0.75 0]');
```

```
    hold on

    subplot(2,3,5)
    plot(t,E,'Color', '[0.75 0.75 0');
    axis([0 60 0 7000]);

    hold on
    subplot(2,3,6)
   plot(t,E,'Color', '[0.75 0.75 0]');
    axis([0 60 0 1]);

end

for j = 1000 : 50 : 1400

   i  = 3.500001;
    tspan = [0, 60];

    options = odeset('MaxStep', 0.01);
    y0 = [j 0 6500 0 0];
    protein = ode23(@kinetics, tspan, y0, options);

    t = protein.x;
    A = protein.y(1,:);
    B = protein.y(2,:);
    C = protein.y(3,:);
    D = protein.y(4,:);
    E = protein.y(5,:);

 hold on

    subplot(2,3,1);
   plot(t, A,'Color', '[0.75 0 0]');

   hold on

    subplot(2,3,2);
    plot(t, B, 'Color', '[0.75 0 0]');

    hold on

    subplot(2,3,3);
    plot(t, C, 'Color', '[0.75 0 0]');

    hold on

    subplot(2,3,4);
    plot(t, D, 'Color', '[0.75 0 0]');


    hold on

    subplot(2,3,5)
    plot(t,E,'Color', '[0.75 0 0]');
    axis([0 60 0 7000]);

```

```
    hold on
    subplot(2,3,6)
   plot(t,E,'Color', '[0.75 0 0]');
    axis([0 60 0 1]);


end

for j = 1400 : 50 : 1800

   i  = 3.500001;
    tspan = [0, 60];

    options = odeset('MaxStep', 0.01);
    y0 = [j 0 6500 0 0];
    protein = ode23(@kinetics, tspan, y0, options);

    t = protein.x;
    A = protein.y(1,:);
    B = protein.y(2,:);
    C = protein.y(3,:);
    D = protein.y(4,:);
    E = protein.y(5,:);

 hold on

    subplot(2,3,1);
   plot(t, A,'Color', '[0 0 0.75]');
   axis([0 60 0 2000]);
   xlabel ('Time')
    ylabel('copy number')
    title('R_i')

   hold on

    subplot(2,3,2);
    plot(t, B, 'Color', '[0 0 0.75]');
    axis([0 60 0 350]);
    xlabel ('Time')
    ylabel('copy number')
    title('R_a')

    hold on

    subplot(2,3,3);
    plot(t, C, 'Color', '[0 0 0.75]');
    axis([0 60 0 7000]);
    xlabel ('Time')
     ylabel('copy number')
     title('C_c')

    hold on

    subplot(2,3,4);
    plot(t, D, 'Color', '[0 0 0.75]');
     axis([0 60 0 30]);
    xlabel ('Time')
     ylabel('copy number')
```

```
      title('C_a')


     hold on

     subplot(2,3,5)
     plot(t,E,'Color',  '[0 0 0.75]');
     axis([0 60 0 7000]);
     xlabel ('Time')
      ylabel('copy number')
      title('C_o')

     hold on
     subplot(2,3,6)
    plot(t,E,'Color',  '[0 0 0.75 ]');
     axis([0 60 0 1]);
     xlabel ('Time')
      ylabel('copy number')
      title('C_o')


end

function dydt = kinetics(t,y)

% defines timing of block pulses
if t < 5
signal = i;
 elseif t < 30
 signal = 0;
elseif t < 35
 signal = i;
else
    signal = 0;
 end

dydt = zeros(5,1);

% reaction rate constants

k1  = 14e-3;
kr1 = 100e-3;
k2  = 25e-7;
k4 = 19500;
k3 = 60e-3;
k5 = 305e-2;

% Hill parameters

n = 3;
theta = 20;


synthesis = k4*y(5).^n ./ (y(5).^n + theta.^n);
decay =k5* y(5);

% differential equations

```

```
dydt(1) = -k1*signal*y(1) + kr1*y(2);           %Ger receptor inactive
dydt(2) =  k1*signal*y(1) - kr1*y(2);           %Ger + germinant active
dydt(3) = -k2*y(2)*y(3) + decay - synthesis;    %closed SpoVA channel
dydt(4) =  k2*y(2)*y(3) - k3*y(4);              %closed and activated SpoVA channel
dydt(5) =  k3*y(4) + synthesis - decay;         %open and active SpoVA channel

end
end
```
